# Sensing of Continuum Robots: A Review

**DOI:** 10.3390/s24041311

**Published:** 2024-02-18

**Authors:** Peter Jan Sincak, Erik Prada, Ľubica Miková, Roman Mykhailyshyn, Martin Varga, Tomas Merva, Ivan Virgala

**Affiliations:** 1 Faculty of Mechanical Engineering, Technical University of Košice, 04200 Košice, Slovakia; erik.prada@tuke.sk (E.P.); lubica.mikova@tuke.sk (Ľ.M.); martin.varga.2@tuke.sk (M.V.); tomas.merva@tuke.sk (T.M.); ivan.virgala@tuke.sk (I.V.); 2 Walker Department of Mechanical Engineering, Cockrell School of Engineering, The University of Texas at Austin, Austin, TX 78712, USA; roman.mykhailyshyn@austin.utexas.edu

**Keywords:** continuum robots, environment sensing, shape sensing, soft robots, flexible sensors

## Abstract

The field of continuum robotics is rapidly developing. The development of new kinematic structures, locomotion principles and control strategies is driving the development of new types of sensors and sensing methodologies. The sensing in continuum robots can be divided into shape perception and environment perception. The environment perception is focusing on sensing the interactions between the robot and environment. These sensors are often embedded on an outer layer of the robots, so the interactions can be detected. The shape perception is sensing the robot’s shape using various principles. There are three main groups of sensors that use the properties of electricity, magnetism and optics to measure the shape of the continuum robots. The sensors based on measuring the properties of electricity are often based on measuring the electrical resistance or capacitance of the flexible sensor. Sensors based on magnetism use properties of permanent magnets or coils that are attached to the robot. Their magnetic field, flux or other properties are then tracked, and shape reconstruction can be performed. The last group of sensors is mostly based on leveraging the properties of traveling light through optical fibers. There are multiple objectives of this work. Objective number one is to clearly categorize the sensors and make a clear distinction between them. Objective number two is to determine the trend and progress of the sensors used in continuum robotics. And finally, the third objective is to define the challenges that the researchers are currently facing. The challenges of sensing the shape or the interaction with the environment of continuum robots are currently in the miniaturization of existing sensors and the development of novel sensing methods.

## 1. Introduction

**Figure 1:**
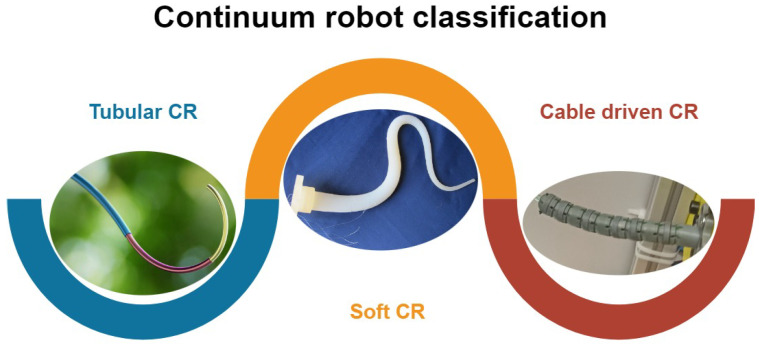
Continuum robot classification.

The progress in the development of biologically inspired robots is eminently forcing various sub-fields of this research to pick up the pace and catch up to this progress. As the development of various types of continuum robots is evolving [1], it is driving the research of new kinematic structures [2], locomotion principles [3], and control algorithms [4] as well as sensors. The continuum robots itself can be classified based on various criteria. One of the most common classifications of the continuum robots divides them into three main groups, being cable-driven continuum robots, tubular continuum robots, and soft continuum robots (see Figure 1). The cable-driven continuum robots are actuated by shortening or extending the length of cables, embedded within the structure of the robot like in [5,6,7,8,9]. By controlling the length of the cables, the position and shape can be controlled. This configuration allows to move the actuators away from the moving part of the robot and leaves more space for the implementation of sensors since the space is often very limited due to the small diameters of the continuum robots. Another type of continuum robot that is studied is so-called tubular continuum robots like in [10,11,12,13,14]. The tubular continuum robots are composed of multiple pre-curved concentric tubes with different diameters, starting with the largest one and ending with the smallest one. The actuation principle of these continuum robots is based on rotating and pushing in and out these tubes in order to navigate to the desired position and achieve the desired shape. One of the main advantages of these robots is their small diameter, which is measured in the order of millimeters. The last group of continuum robots are so-called soft continuum robots as presented in [15,16,17,18]. The main character trait of this group is the soft material that they are made of. The materials are easily deformable, often composed of various silicons and other types of elastomers that upon contact are deformable. This advantage allows them to be used in specific environments where fragile obstacles are present, so that upon contact they do not cause any damage to these objects.

Since these robots are based on elastomers, the most common manufacturing process is based on casting; however, with the increasing trend of 3D printing, 3D printers are used as well. The locomotion principle that these robots often use is mainly based on pneumatic or hydraulic actuation. This means that during printing or casting, specific cavities within the structure of robot are created. Then, these are pressurized, changing the shape and position of the robot according to the different pressures in these cavities. There are also other methods, like the combination of pneumatic actuation and cables or the integration of shape memory alloys and many others; however, the most common methods are using pneumatic and hydraulic principles [19]. Knowledge of how the robot is being actuated and how the kinematic structure of the robot moves and is manufactured is important when selecting the appropriate sensors for sensing the shape or environment of the robot. The type of the sensor and its sensing principle also has to be taken into account when the robot is being designed and manufactured since integration of an inappropriate sensor can negatively influence the properties of the robot.

In this review article, the focus is on the review of existing sensing methods used for continuum robots. These methods are classified on the basis of common principles that they use to detect the shape or contact of the robot with the environment. The paper is structured as follows: First, the methodology of the selection process of the papers is described. Based on the number of selected papers per year, the progress of research is discussed as well. Next, the classification of the methodologies is provided based on the principles that are most often used. These classes are further described based on the selected literature. And lastly, the summary of the paper is provided. The contributions of this paper are in the overview of the current trends in the sensing methodologies that are used for shape and environment sensing of the continuum robots. The classification of these methodologies was also performed to make a clear distinction between the methods. During the study of the presented papers, some ongoing issues were noticed; the authors noticed the lack of generality or universality of the sensors since most of the approaches customize the sensors for their robot due to the mechanical restrictions of the robots. Another issue that deserves to be investigated in the future is the combination of different methodologies or sensing principles. This could possibly bring some redundancy into the measurement, hence improving the accuracy.

## 2. Methodology

To select papers that are relevant and show novelty in the presented research, it is important to select sources that use a thorough review process. This means that the actual relevance of the source is very important. Therefore, the selected papers had to be published by publishers in journals that are either in Scopus or Web of Science databases.

These databases are worldwide databases used by relevant research and academic institutions, and the databases use a thorough peer review process to ensure the quality of the papers. All the papers that were selected from these databases were published between 2010 and mid-2023. Altogether, 52 papers dealing with the sensing of continuum robots were selected and described in this paper.

**Figure 2:**
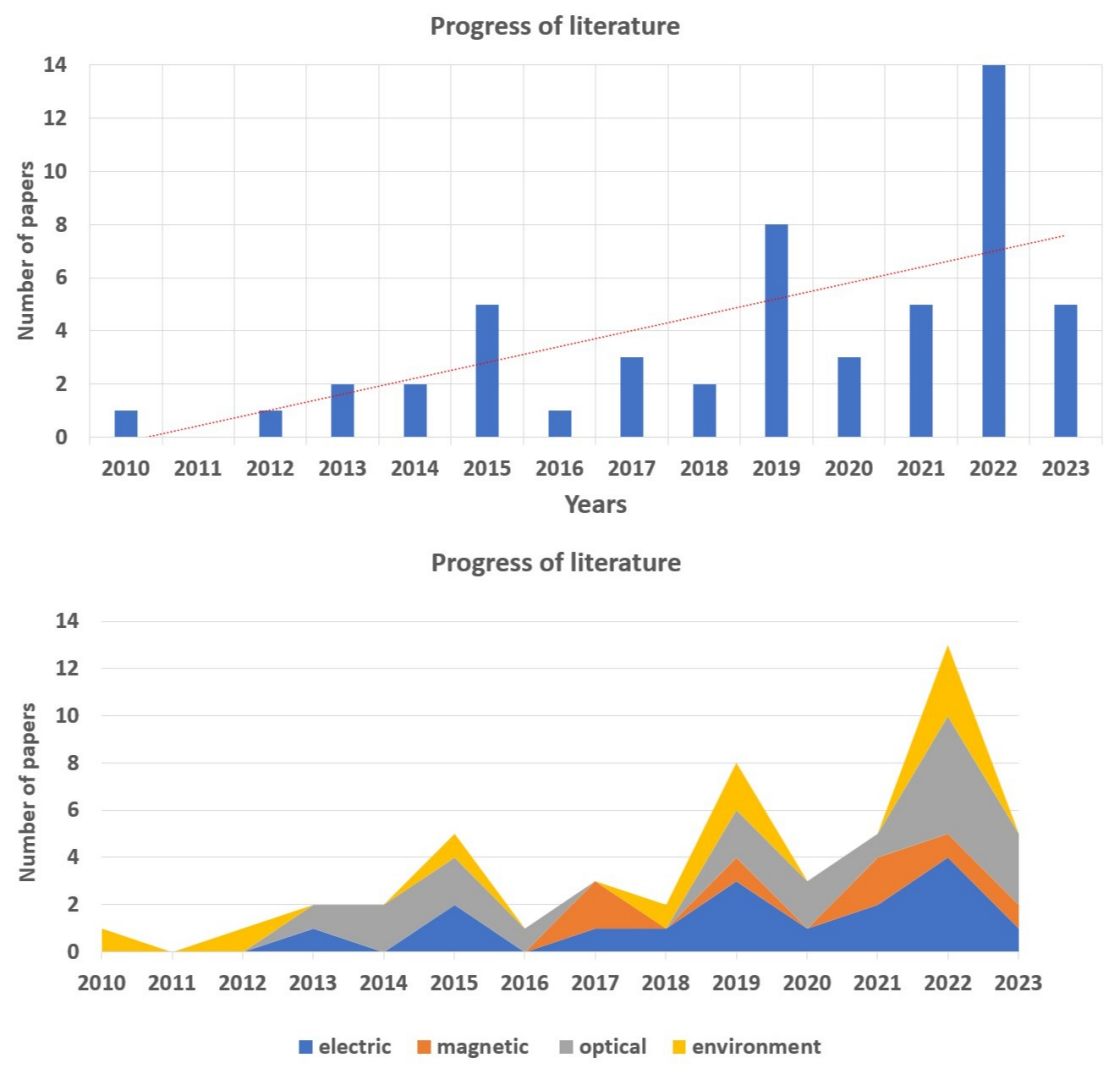
Literature progress from 2010.

These papers were classified based on recurring principles of the sensing methods, as well as the objective of the sensing, that is, what is being sensed. The main areas of focus of these papers were the sensors and the principle that they use for sensing. In Table 1, the summary of papers can be seen. It can be seen that the highest number of papers were published by IEEE publisher at conferences and in IEEE journals that are part of the WoS and Scopus databases. Most papers focused on optical sensors. The overall number of papers suggests that the field of sensing in continuum robots is still developing, and the trend of development of such sensors is increasing. This trend is also supported by the graph in Figure 2. Here, the number of papers per each year is shown based on its category.

**Figure 3:**
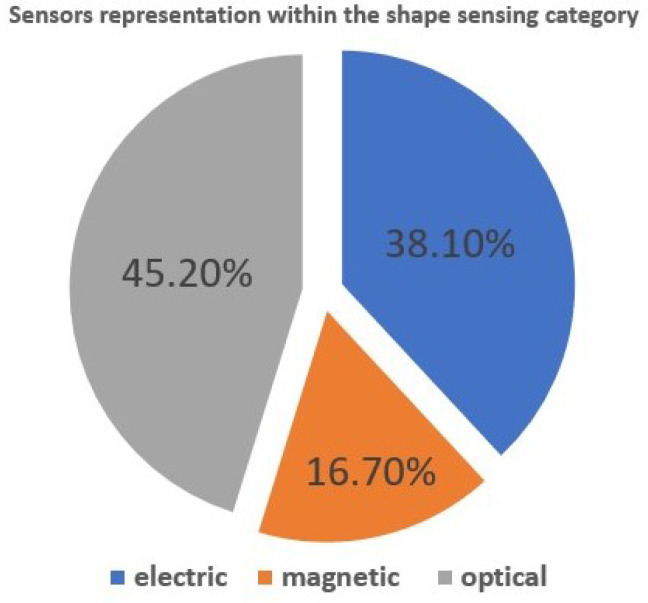
Sensor type distribution within the shape-sensing category.

Overall, the highest amount of relevant papers were found in the category of optical-based sensors, which take up more than 45% of all shape-sensing sensors. In close second are electric-based sensors that take up more than 38%. This distribution is shown in the graph in Figure 3 and is also supported by the bottom graph in Figure 2, where optical and electrical sensors play a significant role, and their trend is increasing with the coming years.

## 3. Classification

The problem of sensing in continuum robotics is facing many challenges. The advantages of continuum robots, such as adaptability, dexterity, and often their small size, have caused problems when interfacing with available sensors in robots. This problem has led to the research of various types of sensors, often proprietary for certain specific robots due to their unique mechanical design and kinematic structure.

**Figure 4:**
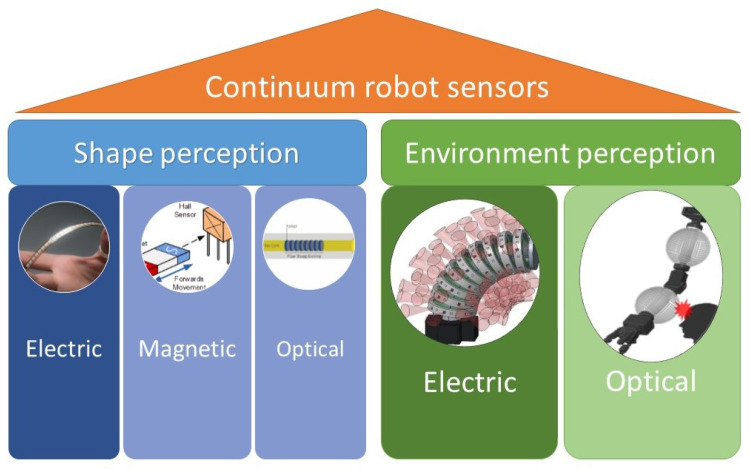
Continuum robots perception classification.

Sensing can be classified on the basis of various criteria, one being the feature that is being observed or sensed by the sensors. In this case, it was either the shape of the robot or the environmental impact on the robot like contacts or collisions with the robot. This is well described in Figure 4.

During the research of various methods that were used for shape sensing, it was noticed that three main principles were used for shape sensing repeatedly. One group of the sensors are sensors that leverage properties of electricity. They measure the change in resistance, capacitance or inductance. This change corresponds to the change in the shape of the robot. These sensors are often integrated in parts of the robot’s body like in [31], or use parts of the robot’s body that have these electrical properties as in [29]. Another two groups of sensors use the properties of magnetism [37] and the properties of traveling light through the optical fibers [45]. When it comes to the sensors that are sensing the environment, the number of papers that were found to be relevant was much smaller but with an increasing trend. This can be seen in Section 2. Hence, the environment perception was classified into two groups. The first group is the electric-based sensors. Just like the shape sensors, these sensors use electrical properties to measure the contact of the robots with the obstacles. The other group of sensors is optical-based sensors. These use the properties of light to sense objects. To get a better understanding of each group of the sensors, the following section will describe the selected papers and working principle of the sensors.

### 3.1. Shape Sensing

The shape sensing of continuum robots is a difficult task. When it comes to rigid-body continuum robots, the limited space is one of the main obstacles in the integration of proper sensing devices into the continuum robots. The shape-sensing sensors for continuum robots generally follow the properties of electricity, magnetism or optical properties to determine the shape of robots. The change in these properties is a result of the change in the shape of the robot. The importance of shape sensing is especially highlighted when it comes to the control of continuum robots. The control without feedback is possible, while an easier solution for control algorithms can be obtained if a constant feedback of the continuum robot’s shape is available. In this section, a description and working principle of three groups of shape-sensing sensors will be described. First, the sensors based on measuring the electrical properties will be described.

#### 3.1.1. Electric Principle

**Figure 5:**
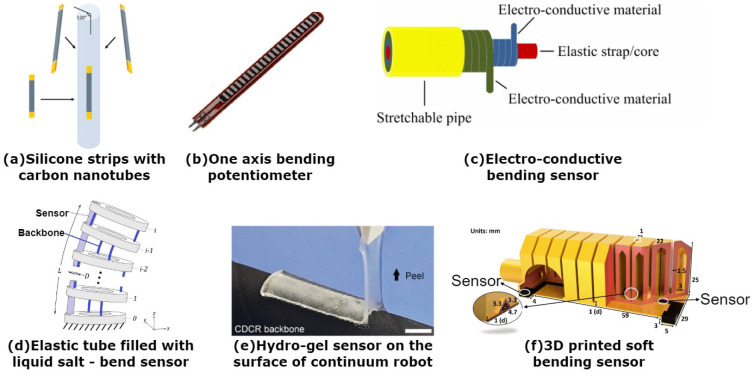
Flexible shape sensors based on electrical properties using the change in resistance during their movement.

One of the most used types of sensors for sensing the shape of continuum robots is that which uses electrical properties to sense the change in the shape or position of the robot. One of these sensors was also presented in [21]. The authors essentially used four sensors equidistantly displaced on the outer diameter of the robot. Each sensor is made of polyurethane mixed with carbon black. Carbon black is a form of carbon with a high surface-to-volume ratio. This substance has a good conductivity, which is the reason for mixing it with elastic material. This mixture of materials produces an elastic strip that changes its conductivity based on the distance of the conductive particles within this material. Therefore, during bending, the authors were able to detect the shape of the robot in two axes. The use of elastic materials with conductive properties was also shown in [22,23]. The authors showed a sensor with an elastic core that has two conductive materials wrapped around in the opposite direction; this can be seen in Figure 5c. Similar to the previous case, based on the bending, the resistance of the sensor is changing, which is detected. By embedding multiple sensors, whose diameter is about 1.5 mm and length about 30 mm, the pose of the robot can be sensed. The maximal sensor stretch is about 156%; up to this point, the data are reliable. An alternative solution was presented in [28]. The authors proposed the integration of commercially available bending potentiometers.

In this case [28], two sensors, like in Figure 5b are opposite each other in the center of the pneumatically actuated soft robot. The main disadvantage of using these sensors is that they can only bend in one axis. Bending the sensor in the different axis would damage the sensor; therefore, the movement of the robot is limited. Another approach utilizing electrical impedance tomography was shown in [24]. As in [22], the combination of flexible material and conductive particles is shown. In this case, the authors opted to combine silicone with carbon fibers that are mixed with the silicone during the molding process. After solidification, the silicone becomes conductive and thus the electrical impedance tomography is used to detect the bending and stretching of the soft material. An electrical impedance tomography is an imaging technology used predominantly in medicine. It uses the conductivity of the materials by applying small alternating current from the electrodes and recording the equipotentials. These are used for the image reconstruction of observed objects. A soft material sensor is also presented in [25]. In this paper, the authors proposed to combine two types of soft silicone, where during the molding process, micro channels are created within the soft material. After the curing process, a conductive liquid is injected into the created channels, and leads are connected. As the material bends, the resistance of the conductive liquid changes, and the bending and strain are detected. On the basis of the experiment, it was found that using two different soft materials with different elasticity provides better sensitivity in detecting the bending of the sensor. The conductive fluid was also used in sensing in [26]. In this case, the authors proposed a soft actuator formed of two layers of polyethylene films. The films are welded together to form a cavity that is filled with saline liquid. In this cavity, multiple electrodes are placed, and electrical impedance tomography is used for sensing the actual bending. An introduction of inductance-based sensors was shown in [34], where a continuum robot based on pneumatic bellows demonstrated the use of such sensors. The sensors are composed of two wires that are coiled around the pneumatic bellow, and based on the movement of the pneumatic bellow, the inductance between the wires changes. An electrical property was also used for sensing in [29]. A derivative of the PneuNet soft actuator was shown with an embedded bendable sensor to measure the bending of the actuator (see Figure 5f). The sensor itself is made by 3D printing using commercially available conductive filaments. This is then embedded in the bottom layer of the pneumatic actuator and bends with it. During the movement, the resistance of the sensor is measured and recalculated to the bending angle of the actuator. To sense the bending of the soft robot, a soft sensor was introduced in [20]. The sensor was manufactured by using silicone and multi-walled carbon nanotubes. The multi-walled carbon nanotubes are a form of carbon in the shape of tubes on a very small scale. In addition, these nanotubes are nested and form larger structures. They have great tensile strength and good conductivity. The carbon nanotubes were combined with silicone to achieve flexibility of the conductive nanotubes. The sensor is in the form of strips placed on the surface of the soft robot so that the shape can be sensed. This is visible in Figure 5a. Another approach is using hydro-gel strips as sensors to sense the bending of the robot [35]. The hydro-gel is a polymer substance that is able to change in response to environmental changes. By using a similar principle as before, as the the robot bends, the resistance of the hydro-gel changes. There are four hydrogel strips stuck to the surface of one segment of the backbone of the cable-driven continuum robot, which can be seen in Figure 5e. A highly stretchable sensor was also presented in [30]. These sensors are in the form of silicone tendons with a channel in the center filled with liquid metal. This material is liquid at room temperature and has good electrical conductivity. The capacitance of the sensor changes with the strain applied on the sensor. Due to the elastic properties of silicone, the sensor was able to stretch up to 600%, and the capacitance changed linearly. Thanks to these properties, the elongation and bending can be measured after implementation into a robot. A stretchable sensor was introduced in [31]. The sensor itself consists of a regular small diameter stretchable spring that is connected in a circuit in which it acts as a variable inductor. The authors then proposed to measure the voltage directly and make the measurement easier. One of the advantages of this method is the calibration curve that was measured before the application to any robot and that is still relevant, therefore showing its universal application possibility. The sensor itself showed low hysteresis and high accuracy. Implementation of liquid-based sensor was shown in [32], Figure 5d. The sensor is composed of elastic tubes that are filled with liquid salt. The sensors were presented in a two-section continuum robot driven by cables. Each section had two sensors that were placed on the circumference of the robot, and based on the change in the resistance, were able to detect the bending angle of each section. The combination of mathematical modeling and information from sensors was shown in [33]. The presented paper combines a piecewise constant curvature mathematical model and information from IMU sensors that are attached at the bottom and top of the robot. The IMU sensors, or Inertial Measurements Units, are sensors capable of measuring acceleration and angular velocity. These sensors are great for integration in the robots since they are relatively low cost and have a small size [72]. By combining the information from the sensors and mathematical model, the mean pose estimation error achieved was 2.7 cm. By using stretchable fabric coated with conductive polymer, a prototype of the sensor was introduced in [27] for the shape sensing of soft robots. The sensor consists of two electrodes that sandwich multiple layers of this conductive fabric. By applying force on this sensor, its resistance changes accordingly. Stacking multiple layers of fabric increases the range of the measurement; however, it takes longer to detect the change, and therefore the balance between a sufficient number of layers and response time is needed.

The presented papers followed the sensors that sensed electrical properties in order to determine the shape of the robot. They were implemented either in the robot or were actually part of the robot bodies themselves. The most obvious advantage of using these sensors is the relatively low cost of the necessary materials and equipment. It is also quite convenient to work with the signal from the sensors. As one of the disadvantages, the disturbance in the signal can be caused by other electrical devices in the proximity of the sensors, or if part of the body acts as a sensor, after the contact of some other conductive object in the environment, it can cause problems during the sensing. Another category of shape-sensing sensors are sensors based on sensing magnetic properties.

#### 3.1.2. Magnetic Principle

**Figure 6:**
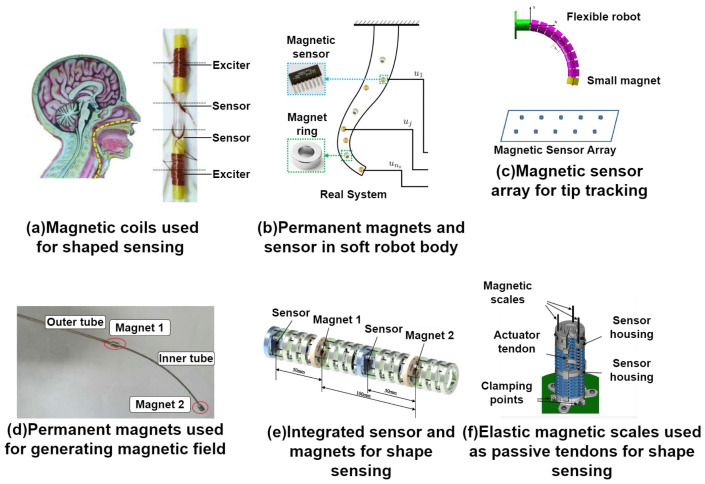
Shape sensors based on magnetic properties using permanent magnets and and electric coils.

Along the electrical properties, the magnetic properties are often used to track the motion, position and orientation of the objects. This is also used in continuum robotics. The authors in [36] introduced a study in which a two-segment concentric tube robot uses magnets to track its position. The robot is visible in Figure 6d. A magnet is placed at the end of each segment, and it is tracked by a magnetic tracking system composed of an array of sensors. The array tracks the 3D positional data, as well as the 2D orientation data, based on the intensity of the magnetic field generated by the magnets on the robot. By measuring these two points and by using the Bézier curves, a shape reconstruction algorithm is then used to reconstruct the shape of the robot. The same method was also presented in [37]. In this study, the authors applied the same approach on a single-segment cable-driven flexible continuum robot that can be seen in Figure 6c, whose movement was restricted to 2D space. So, again, the magnet was mounted on the tip of a continuum robot segment, and the same shape reconstruction algorithm was used. In [40], the authors also presented an approach using permanent magnets. The approach was showcased on a cable-driven continuum robot, which is shown in Figure 6e. In one segment, two permanent magnets were placed in the structure of a robot with two sensors sensing the density of the magnetic flux during the movement of the robot. To determine the position of the sensors and magnets during the movement of the robot, the superposition principle was used on the measured magnetic flux densities in order to get rid of the noise in the measured data. The results show that bending up to 90 degrees is measurable; however, the maximal and minimal measured error is roughly 9 mm apart. The application of magnets was also shown in [39] on a multi-arm cable-driven continuum robot.

The authors placed magnets at the end of each robot and performed movements in front of a sensory array. The sensory array then tracked the magnetic field of each magnet to determine the position of each robot. Since the robot is independent of the sensory array, there are two coordinate systems, one of the robot and one of the sensory array. In order to perform the translation, a motion capture system was used to obtain the information about the relation between the coordinate systems. This system only tracks the tip position; however, this information can be used for shape estimation. Instead of permanent magnets to produce magnetic field, coils can be used like in [38]. This can be well seen in Figure 6a. Here, the authors demonstrated the use of coils on a plastic tube catheter. Along the length of the tube, the excitation coils were wound on the tube, between which the sensing coils were placed. There were two sensing coils in between every two excitation coils. Each of the sensing coils was wound in an inclined plane facing each other. This was performed so that one coil would sense the bending in the x-y plane and the other in the x-z plane. Excitation coils are powered and create a magnetic field; this field is then detected by sensing coils. When a tube changes its shape, the inductance of the sensing coils changes, which is measured in the change of the voltage of the sensing coils. Based on this, the shape of the tube can be observed. In [41], the authors proposed an integration of permanent magnets and magnetic sensors into the soft robot (see Figure 6b). The magnets were axially symmetric, and placed in the center of the robot. A total of three magnets were placed in the three-segment soft robot as well as five sensors, which were randomly placed in the robot structure. The proposed algorithm in this paper focuses on the shape reconstruction of the robot by estimating the shape of each segment based on the input from the magnetic sensors. This is achieved through the training of the sensor measurement predictor (neural network), which is trained on the result from mapping the kinematic relationship between the sensors and magnets, and the output of the predictor is the sensor measurement. In [42], the authors proposed to use two linear Hall effect sensors and elastic magnetic scales. The magnetic scales were implemented in a cable-driven continuum robot, from Figure 6f, acting as passive tendons, so they adjusted to the shape and movement of the robot. The Hall sensors are in pairs, and they measured the distances between the sensors in order to calculate the shape and tip position.

These sensors were based on the measurement of magnetic properties to measure the shape of the continuum robots. The pros of these sensors might be the use of permanent magnets or coils that can be miniaturized quite well, and then the use of external sensors to track the changes in the sensed property. However, the presence of other ferromagnetic materials might influence the sensory reading and cause false interpretations of the measured data. The last group of shape-sensing sensors is based on principles of traveling light through optical fibers and vision systems. Leveraging these properties has the advantage of resilience against electrical and magnetic disturbances from the environment and surrounding devices.

#### 3.1.3. Optical Principle

**Figure 7:**
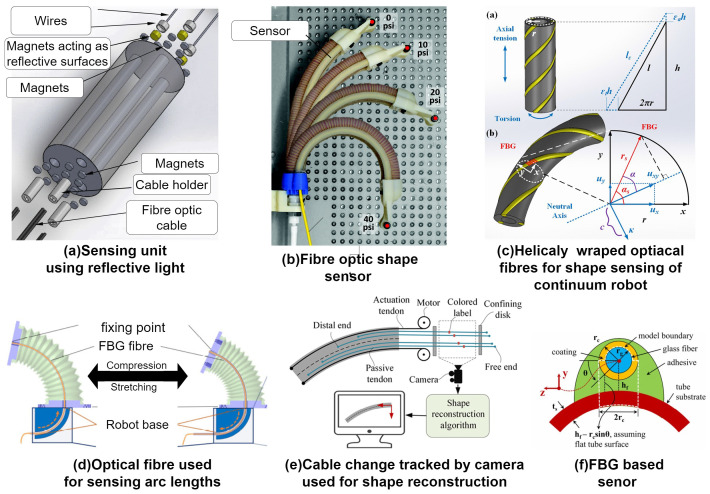
Shape sensors based on sensing the properties of light using the optical fibers.

Another principle that is often used for measurement is the optical principle, which is leveraging the properties of the traveling light particles. The use of these properties is often advantageous where electrical or magnetic properties might be influenced by the environment or other devices nearby. One of these sensing principles was introduced in [43]. The sensor was used for sensing the bending of a soft flexible manipulator, which consisted of a sensing cylindrical unit and soft bending unit on top. The bending unit can be seen in Figure 7a. In both of the units, three symmetrical channels were made around the central axis. At the bottom end of the sensing unit, transmitter and receiver fiber optic cables were placed. In the bending unit channels, wires are placed, which adapt to the elongation and bending of the unit. At the end of the wires that are in connection with the sensing unit, a magnet is connected to every wire. As the robot is bending, the magnets are moving according to the displacement of the robot, and the distances in the channels between the magnets and optical fiber ends are changing. The magnets act as reflective surfaces to the emitted light, and based on the change in the reflected light intensity, the length of each channel is measured and the shape is calculated. The use of optical fibers was also utilized in [44] as well as [45]. In [45], the authors proposed to use three optical fibers along the length of a multi-segment continuum robot.

Optical fibers were evenly placed around the robot’s body and attached with the glue. This can be seen in Figure 7f. The sensing technique utilized in this paper is called fiber Bragg grating (FBG). The optical fibers are grooved every few centimeters. These grooves cause the light to reflect during the bending of the optical fibers. Three optical fibers are then used to measure the bending in these points, where grooves are made. The FBG principle of bending measurement was also introduced in [46]. In this case, a cable-driven continuum robot was used to test the sensors. The robot was driven with two pairs of cables and was able to move in one plane. The optical fibers used for sensing were placed in between the pairs of cables, forming a triangle formation with the cables. Based on the performed experiments, the curvature sensing seems to be more accurate for larger curvatures; however, the average error of 3.14% suggests a good enough performance for the majority of use cases of the continuum robots. A similar system using optical cables for sensing was used in [47]. The paper presented the design of a curvature-sensing system for a continuum robot. The sensory system senses the bending of the robot using light intensity modulation. The presented continuum robot consists of two flexible units that are driven by the cables. Units also contain passive cables, which are optical cables and are used for sensing the shape of each unit. Again, two fibers are used, one as an emitter and one as the receiver. There are multiple couples of these cables collocated in each segment of the robot. As the segment bends, the distance between the sensing pair changes, and the bending of the robot is sensed. The presented method shows only six degrees of error in the measurement compared to the real bending of the robot. The FBG sensing was also used in [49]. In this paper, the authors used the optical fibers to measure the bending as well as force. By embedding the optical fibers in helical grooves on the surface of the continuum robot like in Figure 7c, the sensing of the strain is used in a force–curvature strain model. It maps the relations between the measured strain from FBG sensors and the tip force of the robot. Based on these measurements, the aforementioned model shows the correlation between the strain and curvature of the robot. The implementation of optical cables in the robot was shown in [50]. The robot is composed of two discs connected with spring and four cables around the spring for actuating the robot. The sensors are composed of Nitinol spring and optical cable embedded in a silicone cylinder. Nitinol is a nickel–titanium alloy, which has a great shape memory effect and a superelasticity effect. Thanks to these properties, the spring can withstand much more deformation cycles and return to its original shape unharmed. The optical cable is approximately half the length of the robot segment, and the other half is the Nitinol spring. The optical fiber is in series with the Nitinol spring so that the tensile force applied on the robot can be measured. The force applied on the robot, causing it to bend, is translated through the Nitinol spring to optical cables, shifting the wavelengths of the reflected light in the optical fiber. Based on this measurement, the bending of the robot can be estimated. In [56], the authors focused on the implementation of a commercial shape-sensing multi-core optical-fiber-based sensor. The FOSS sensor was implemented in the design of the robot (see Figure 7b), following the outer shape of the robot. The sensor is able to detect bending and twisting along its length every 0.8 mm and with a sampling rate of 250 Hz. The sensor is capable of measuring the shape, bending and twisting of the sensor; therefore, it is irrelevant to the structure of the robot. The positional accuracy and twisting accuracy during measurements were submillimeter. The implementation of FBG sensors was also used in [53]. The optical fiber was incorporated within the proposed robot. The fiber measured strain, which was then used to calculate the actual shape of the robot. A similar case of sensing the shape of a catheter robot was shown in [57]. The FBG optical fiber in this case is forming a triangular structure together with two Nitinol wires. By optimizing the distance between the optical fiber and neutral plane of the sensor, the range of bending angles can be expanded. By implementing multiple sensors in the catheter robot, the shape of the robot is obtained. Another implementation of FBG sensors was also shown in [58]. The sensor was helically wrapped and incorporated in the design of a pneumatically controlled soft manipulator. The soft material that was used allows the elongation of the robot by about 56%, while the sensor is able to adjust to the change since it is helically wrapped around the robot. This change in the shape or elongations also cause a change in the wavelength shift based on the shape of the robot that can be reconstructed. Another instance of using optical fibers for sensing was introduced in [54,55]. The principle used in this paper is building on the traditional FBG; however, it uses multi-core fiber draw tower grating, or DTG. This fiber contains multiple cores, which allows for higher resolution of the sensing. The fiber contains four cores and sensing nodes every 15 mm. The sensor passes through the robot and moves freely, not affecting the robot. The overall structure of the robot is sensed by a single DTG sensor, where every node is measuring the strain based on the change in the wavelength of the light passed through the sensor. Based on the measured strain, the curvature and shape are reconstructed utilizing the Bishop frame and the corresponding relations between the curvature and strain. Another implementation of the FBG sensors and the shape reconstruction algorithm was shown in [59]. In this case, the author used a sensing rod. This rod consists of two optical fibers glued to the sensing rod in order to avoid the influence of the temperature by using temperature decoupling. There are five sensing points along the length of the sensing rod that are used for the shape reconstruction. The shape reconstruction algorithm combines linear and cubic spline interpolation in order to define the relation between the curvature and arch length. In [48], the authors proposed an FBG-based sensor in combination with variable length and curvature measurement of a soft robot with the ability to not only bend but also extend; this robot can be seen in Figure 7d. The optical fiber is integrated in the body of the soft robot and within a rigid channel in which it is allowed to move freely. The channel is fabricated with a specific bend, which is used for sensing the extension of the robot. As the robot is extending or contracting, the fiber is moving with the robot and moving through the rigid channel, and the particular grating in the fiber can be tracked, and the length is measured. The FBG type of sensing was also used in [60]. The authors used multi-core optical fiber for shape reconstruction of the continuum robot. The shape reconstruction model is based on an extended Kalman filter, combining the sensory signal from an FBG sensor and approximate kinematic model of a continuum robot. The drawback of inaccurate models is improved with the signal from the sensor, and the shape estimation is more accurate. The Kalman filter is a popular algorithm used for the estimation of unknown variables based on measured data; therefore, this is used in this application for combining the measured signal and approximate kinematic model to produce the shape estimation. Another approach using optical fibers is shown in [61]. The authors pulled two optical fibers through slots in the structure of a continuum robot. On one end of the optical fiber, a commercially available LED was used as a transmitter, and on the other end, a receiver was placed. As the robot moved, the transmitted light intensity changed; the more the robot bent, the more the light intensity decreased and the more the light was lost. A different approach leveraging optical properties is shown in [51]. Here, the authors used passive cables with markers and cameras, which can be seen in Figure 7e. The cameras were used for tracking the change in the position of the markers as the robot moved and the passive cables with it. This information was then used for shape reconstruction using screw theory and material frame. Another approach using cameras is [52]. In this paper, the authors used an RBG camera in a fixed position from the robot. The frames from the camera were fed to a MoSSNet neural network to reconstruct the shape and orientation of the captured continuum robot.

When it comes to soft and continuum robots, the optical sensory system is one of the most commonly implemented sensors. Their advantages, like low weight, flexibility and resiliency against the influence of environmental disturbances, make them a suitable choice for implementation within a robotic system like continuum and soft robots. One of the disadvantages can be the price of the materials and equipment needed for optical fibers and for light-emitting sources and receivers. These three groups sense the shape of the robot, not taking into account the environment around the robot.

### 3.2. Environment Sensing

Another class of sensors is the sensors that detect the environment and its interaction with the robot. These sensors are used typically when working on the motion planning and task planning of robots. It is necessary to follow the interactions with the environment and obstacles and take them into account during the movement and planning. The category of these sensors is not as developed as shape-sensing sensors; therefore, the number of found papers is much lower. In this category, there are two groups of sensors defined based on their principle. One is electrical-based sensors, using the change in some electrical property of the sensor, and the other one is optical-based sensors, using the properties of traveling light.

**Figure 8:**
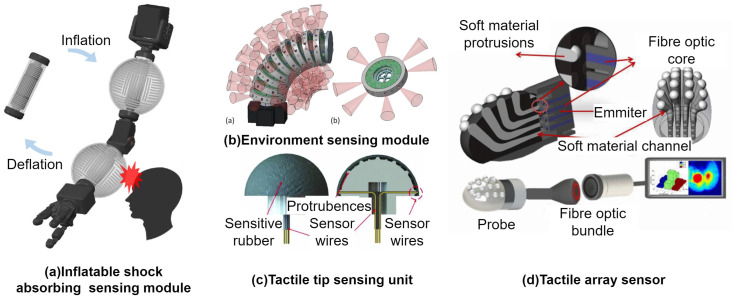
Environment sensors: electric-based sensing (**a**,**c**); optical-based sensing (**b**,**d**).

The electrical-based sensor uses the properties of electricity that have some correlation with the contact of the robot and an object. These sensors are often embedded in the outer layer of the robot. This approach was also used in [63]. Here, the authors proposed a sensor module acting as a skin that has embedded micro-channels filled with a conductive liquid. This can be seen in Figure 8a. The modules are inflated, and upon contact, the electrical resistance changes. One of the advantages of the module is the softness of the used material. The material used for the skin is made of soft and stretchable elastic material, which upon contact acts as a damper and not only senses but also acts as a shock absorption element.

This can be very well seen in Figure 8a, where these modules are used as part of a robot arm. Upon contact with humans, they are able to sense the contact as well as absorb the shock from the collision. A soft dome structure was introduced in [69,70] to detect an object in front of a robot. This structure contains an array of piezoresistive tactile sensors, which upon contact change their resistance. This sensor can be seen in Figure 8c. The proposed method uses the electrical impedance tomography principle to detect the contact position on the dome. Due to the shape of the dome, the contact location is positioned in 3D space; hence, the classical 2D image has to be converted to 3D, and the direction of the contact is in the form of a vector from the center of the sensor to the touch point [70]. A different approach was shown in [71], where a continuum robot for search and rescue was developed. The robot consisted of multiple segments, where each segment contained various sensors. The IMU sensors are used for shape estimation of the robot, and microphones are used to localize the survivor. For environment sensing, the piezo-resistive vibration sensors are used as contact detectors. In addition, a high-speed camera placed in front was used to map the environment using the SLAM algorithm. Simultaneous localization and mapping (SLAM) is a technique used in robotics to map the environment and localize the robot in it [73]. In [68], the authors used load cells for sensing forces acting on the backbone and an external tracking of the tip of the robot. In this paper, two methods for contact detection were researched. One is reliant on a static model of the continuum robot and measuring the deviation in the joint forces, which implicates the contact of the robot with an obstacle or an object. Another approach is using the kinematic model of the robot with constraints to estimate the contact location. It leverages the fact that from the point of contact, the robot’s bending and shape are changed, and therefore it is possible to detect the location of the contact. Similarly, in [65], the authors use screw theory to compute the screw motion deviation. The collision is then detected by watching the aforementioned deviation, which is the difference between the expected screw axis, calculated from the kinematic model without any obstacles, and the actual screw axis of motion is based on information from the magnetic-based sensors sensing the endpoint of each segment.

The other group of environment sensors use the properties of traveling light to detect either an object or contact with the object and robot. In [66], to detect contact, embedded optical fibers were used in soft material. The outer layer was covered with an array of soft bumps that are hollow from the inside and connected with channels to the optical fibers; see Figure 8d. For each channel, there is a pair of optical fibers, one is the emitter and the other the receiver. As the bump comes in contact with an object, it is deformed. This causes the light emitted from the optical fiber to travel a smaller distance, hence detecting contact with an object. Another approach of using a sensory array is presented in [67]. In this case, the authors proposed a continuum robot consisting of multiple segments (see Figure 8b). Each segment contains multiple sensors for sensing the environment around the robot as well as the force. Each module is composed of multiple time-of-flight sensors to detect an object and map the environment as well as Hall effect sensors and magnets to detect contact and force [64]. A data-driven method for contact localization is presented in [62]. The approach presented in the paper uses a machine learning technique called AutoEncoders [74], which is a type of neural network. This method leverages the shape sensing of the robot, which is detected by FBG, and the AutoEncoder is used to detect the contact based only on these data, which are collected during the movement of the robot without contacts. The author’s approach is focusing on the disturbance in the tip estimation error of the robot, which indicates the contact location.

The environment sensors are a group of sensors that focus on the detection of obstacles or objects that might collide with the robot. The variety of sensors range from electrical sensors that detect collision to optical sensors that can detect the object before the collision happens. The use of such sensors is important in the path-planning phase when the knowledge of the environment is necessary. The advantages and disadvantages of certain types of sensors might be dependent on the application that the robot will be used for. In some cases, contact with the obstacles might be completely prohibited; therefore, sensors that map the environment before the contact are necessary. In other cases, cheaper and simpler sensors or solutions might be adequate for the case.

### 3.3. Applications

**Figure 9:**
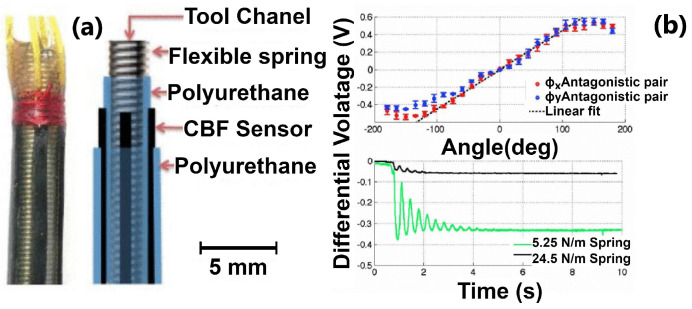
Continuum robot endoscope with electric-based shape sensor: (**a**) relation between the measured voltage and angle of the bending actuator; (**b**) step response of the tested sensor.

The applications of continuum robot sensors are in sensing the continuum robot’s shape or environment. These sensors can be best represented by case studies of these continuum robots. One of these sensors can be seen in Figure 9. This sensor is an electric-based sensor made of polyurethane mixed with types of carbon particles in the form of stripes.

Four stripes are evenly placed around the central backbone of a continuum robot to sense the bending of the robot. In Figure 9a, the relation of the angle and measured voltage can be seen, and in Figure 9b, the step responses tested on springs (central backbone of the robot) with different stiffness. The intended application of this robot is in medicine, to be used as an endoscope with various instruments and cameras. The design of the sensor is scalable, and therefore the potential use of various types of continuum robots in surgical robotics is possible.

**Figure 10:**
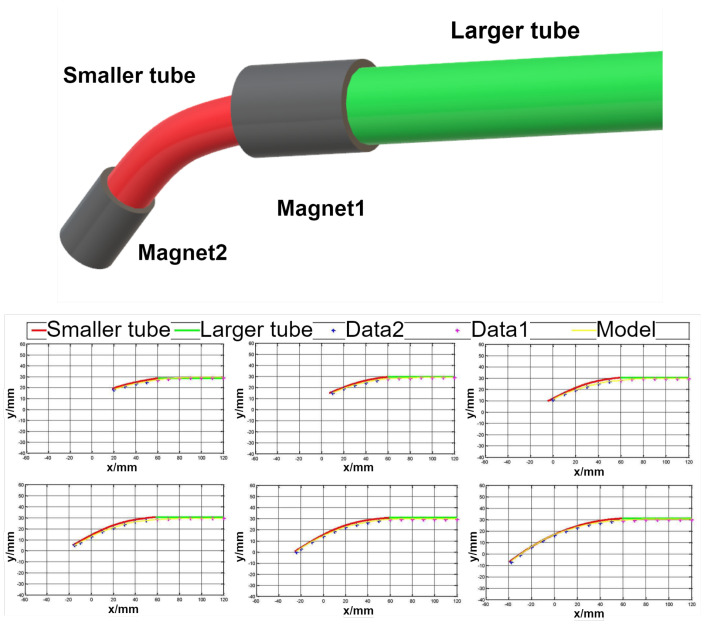
Tubular continuum robot demonstrating magnetic-based sensors for shape sensing; measured data during different stages of the movement.

Another case study is shown in [36], where the authors demonstrated the use of magnetic-based sensors. This can be seen in Figure 10. In this study, a tubular continuum robot with two sizes of tubes is used, and each tube has a magnet attached at its end. These magnets are then tracked by an external array of magnetic sensors.

Each of the magnets generates a magnetic field which is tracked, and by using Bezie curves, the shape of the robot is reconstructed. The graphs of the reconstructed shape based on the magnet tracking can be seen in Figure 10. These graphs show the tubular robot in various stages of the robot’s movement. The use of permanent magnets and external sensors has advantages in environments that do not contain any ferromagnetic objects or materials that could possibly influence the measurement. Such a use case could be minimally invasive surgery, where tubular continuum robots are often used. When it comes to minimally invasive surgery, it is important to keep the size of the robot as small as possible; therefore, the use of this kind of setup with external sensors can be beneficial to keep the size small.

**Figure 11:**
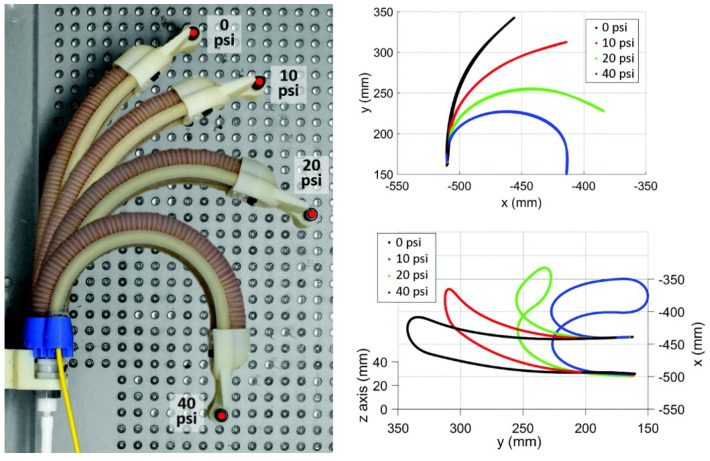
Soft flexible actuator incorporating commercial optical based sensor used for shape and tip tracking; measured shape during experiments.

A case study showcasing the optical-based sensors is shown in [56]. The authors used an optical-based sensor, based on a multi-core fiber optic cable. The sensor was included in the design of the soft robotic actuator, the sensor being placed along the length of the robot as well as at the tip, forming a continuous line, which can be seen in Figure 11 (white line along the robot).

The sensor itself allows for the measurement of bending and twisting every 0.8 mm. Multiple experiments were carried out by the authors, testing the shape measurement and twisting of the robot. These experiments can be seen in Figure 11, where graphs show the measured shape of the robot during the various actuation tests. The sensor was implemented and tested on a soft actuator, with the possibility of implementation in different continuum robots.

**Figure 12:**
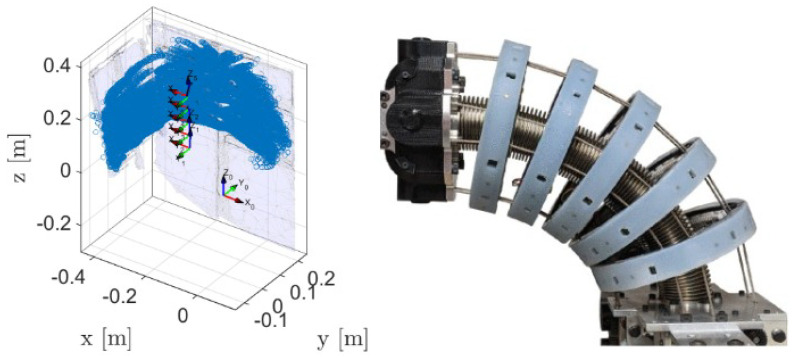
Implementation of time-of-flight sensor for environment sensing; map of the environment using ToF sensors.

The application of environment sensors is showcased in [64,67]. In this study, the authors proposed a custom continuum robot arm, made of discs containing environment sensors (Figure 12). In this application, each disk has eight ToF sensors and Hall effect sensors with magnets. The Hall effect sensors and magnets are used to measure the external force applied on the robot. The other type of sensors is ToF, or time-of-flight, sensors. These are based on measuring the distance to a object using the time of light traveling to and from an object.

The eight evenly placed ToF sensors in each disk scan the environment around the robot and are able to determine the location of the objects around the robot. The intended application of this robot or these sensors is in human–robot collaboration tasks, where environment perception is a key factor.

## 4. Conclusions

In this article, environment and shape-sensing sensors were reviewed and described. In every section, the sensors were described chronologically from the oldest to newest in each category. As seen in Section 2, Figure 2, the research of sensors is picking up the pace, and more and more papers are dealing with sensing in continuum robots. It is also visible from the bottom graph in this figure that optical sensors are the most dominant group of sensors, closely followed by electrical-based sensors. Environment sensors focus on the contact detection using various methods based mostly on the application of sensors on the surface of the robot so that in the case of contact, the sensor would detect the obstacle; however, some sensing techniques use the deviations of the robot’s shape from the expected shape to detect the contact location.

The shape-sensing sensors were divided into three main categories: electric-based sensors, magnetic-based sensors, and optical-based sensors. Each group of sensors predominantly measures the change in the properties of electricity, magnetism or optics, respectively. These changes then reflect the change in the shape of the robot, which is tracked. When it comes to electric-based sensors, they often focus on the change of resistance or capacitance in various types of flexible sensors or the robot body itself. In order to measure this change in resistivity, electrically conductive particles had to be present in the material that was used for manufacturing the robot’s body. The advantage of using such sensors is the low cost of materials and equipment necessary for measurement. The disadvantage of using these kinds of sensors can be, in some cases, the analog signal of these sensors. This signal can be influenced by the external devices.

Another group of sensors focus on the use of magnetism to track the robot’s shape. These sensors use permanent magnets or coils to generate a magnetic field or flux and track it with external sensors. These data are then used to reconstruct the shape of the robot. One of the advantages of using these sensors is the ability to miniaturize the permanent magnets or coils and integrate them into the robots directly and track them with the external sensors. The problem of tracking these magnetic properties comes up only when other magnetic materials might occur in their presence and influence them.

Lastly, optical-based sensors mostly focus on the use of optical fibers and FBG sensing. The FBG sensing technique is using the optical fibers with a number of segments where the refractive index is periodically changed, and this creates a specific wave. By putting a strain in the spots where the refractive index is changed, it is possible to measure this change in the strain acting on the optical fiber and reconstruct the shape. Another sensor in this group uses shape reconstruction algorithms based on the data from cameras.The resilience against external disturbances is the biggest advantage of using optical fibers for sensing. The disadvantage can be the higher cost of materials and equipment that is necessary for the measurement.

Environment-based sensors are used to detect collisions or obstacles before the collisions. These sensors use various techniques to sense these interactions. Two main groups of sensors were described in this paper. One group is based on measuring the electrical properties in order to detect the objects in the vicinity or during the collision with the robot. The other group of sensors use properties of light to detect the obstacles and map the environment and the interactions of the robot with it. The main advantage of using these sensors is in the phase where the path is planned and the obstacles influence the robot’s trajectory.

There were three main objectives of this review paper. Objective number one was to clearly categorize the sensors and help the reader to gain an insight in an area of continuum robot sensing. This was clearly defined in Section 3 as well as in Figure 4, where sensors are classified into shape- and environment-sensing sensors, and these groups were further divided and described based on the physical phenomenon that they were sensing. Objective number two was to determine the trend of the research of continuum robot research and how the field is progressing. This objective was discussed in Section 2, where the increasing trend of research papers was identified. The total number of selected relevant papers was 52. The most used type of shape-sensing sensors were based on the optical principle, taking up to 45% of the selected papers. It was also identified that the amount of optical- and electric-based sensors will be increasing with the coming years. Objective number three was to identify the current challenges that the research of continuum robot sensors is currently facing. As mentioned in the previous sections, the disadvantage of many of the reviewed sensors is lack of universality, meaning that the sensors are often implemented in the continuum robot in a way that is specific to that precise design of the robot. This disadvantage makes it hard to implement one type of sensor in a variety of continuum robots. Other challenges like miniaturization and the combination of multiple sensing methods are also important and deserve focus in future research.

The field of continuum robotics is a perspective field that is rapidly developing. With the development of new kinematic structures and locomotion principles, the need for sensing the shape and environment interaction is increasing. This need is driving the research of novel methods of sensing and novel designs of sensors. The future research in this area should further focus on sensing methods and new designs of sensors. The miniaturization of sensors and their implementation into the continuum robots is an important step in the research as well.

## Figures and Tables

**Table 1:** Selected papers for review.

Perception	Sensing Technology	Paper	Publisher	Type of Paper
Shape		[20]	AAAS	Journal
		[21,22,23,24,25,26,27]	IEEE	Conference
	Electric	[28,29,30,31,32]	IEEE	Journal
		[33]	Springer	Conference
		[34]	Springer	Journal
		[35]	Wiley	Journal
		[36,37,38]	IEEE	Conference
		[39]	IEEE	Journal
	Magnetic	[40]	Elsevier	Journal
		[41]	RCS(WoS)	Journal
		[42]	MDPI	Journal
		[43,44,45,46,47,48]	IEEE	Conference
		[49,50,51,52,53]	IEEE	Journal
		[54,55]	IEEE/ASME(WoS)	Journal
		[56]	Mary Ann Liebert(WoS)	Journal
	Optical	[57]	EMERALD(WoS)	Journal
		[58]	Wroclaw univ science technology (WoS)	Journal
		[59]	Springer	Conference
		[60]	Springer	Journal
		[61]	MDPI	Journal
Environment		[62,63,64,65]	IEEE	Journal
		[66,67,68,69]	IEEE	Conference
	-	[70]	EMERALD(WoS)	Journal
		[71]	Springer	Journal

## Data Availability

Data are available in the manuscript.

## Abbreviations

The following abbreviations are used in this manuscript:

CR	continuum robot
IEEE	Institute of Electrical and Electronics Engineers
FBG	fiber Bragg grating
DTG	draw tower grating
WoS	Web of Science
